# Categorizing music by genres

**DOI:** 10.1111/nyas.15404

**Published:** 2025-07-09

**Authors:** Elke B. Lange, Emily Gernandt, Julia Merrill

**Affiliations:** ^1^ Department of Music Max Planck Institute for Empirical Aesthetics Frankfurt/M Germany; ^2^ Institute of Music University of Kassel Kassel Germany

**Keywords:** categorization, genre theory, heuristics, liking ratings, musical taste

## Abstract

In arts and music, exemplars are categorized into genres, but those are not static and change dynamically. In musical taste research, there are strong differences in number and width of genre categories, and it is unclear how category width (broad genres vs. narrow subgenres) affects liking evaluations. The use of broad genre labels has been strongly criticized. We address this issue by a quantitative approach, comparing liking evaluations of 15 musical genres with 100 embedded and two nonexistent subgenres (*N* = 804). We applied a wide range of analyses (correlations, regressions, random forest modeling, factor analyses). Results converge, showing that evaluations of the majority of nested subgenres were highly similar to the related genres. The nonexistent subgenres revealed evaluation heuristics, enhancing consistency. For some genres, liking of subgenres grouped based on historical (traditional–modern), cultural (German subcultures), or functional (danceability) reasons. The genre labels *pop* and *rock* were less appropriate. We provide a list of 24 genres to assess musical taste sufficiently for general applications. Importantly, the concept of genre for taste studies is still useful.

## INTRODUCTION

Categorization is one of the most basic cognitive processes, supporting fast and suitable reactions to the perceived world by providing structured information.^1^, ^2^ Categories differ in width from broad to narrow and are often hierarchically nested (e.g., vehicle, car, BMW). Different from natural objects, in art, genre classification is applied in a more complex and dynamic way, depending on historic time periods, language usage and conventions in specific discourse communities,^3^, ^4^, ^5^, ^6^ influenced by the publishing industry,^6^, ^7^, ^8^ and interrelated with social conditions,^8^, ^9^, ^10^ making genre boundaries fuzzy, ambiguous, and subjective.^11^

Nevertheless, in empirical studies, genre terms have to be used to define and communicate the research subject. Despite similar questions, the compiled genres differ considerably, ranging from small to large sets (e.g., assessing musical taste by 7 (Ref.12) or 104 (Ref.13) genres), and from broad genre labels (e.g., *rock*) to narrow genres (e.g., *soft rock* and *hard rock*), and subgenres (e.g., *rock'n’roll, punk, indie, alternative rock*). Broad categories might not be differentiated enough to cover taste sufficiently well^14^, ^15^ or to be sociologically meaningful.^16^, ^17^ A large, detailed range of narrow categories, however, takes up much time during assessment, causes missing data,^13^, ^18^ and increasing the number of categories for the same semantic space increases the difficulty to differentiate between them.^19^ The question arises as to which category width is sufficient as well as practicable. Research from computer linguistics and computational semantics have addressed such questions by investigating large data sets of annotated images and sounds (i.e., ImageNet,^20^ AudioNet^21^), or lexical databases (i.e., WordNet^22^).

We focus in our research on category width of musical genre in the context of taste research, but our findings may also relate to other domains (e.g., from [liking] evaluations in the arts to everyday objects). Musical taste has been investigated in many different contexts, showing relations to personality,^13^, ^18^, ^23^, ^24^, ^25^, ^26^, ^27^, ^28^, ^29^, ^30^, ^31^ political orientation,^26^ self‐esteem,^32^ gender,^33^, ^34^ age,^35^, ^36^, ^37^ or psychological disorder.^12^, ^38^, ^39^ That is, musical taste relates to who we are.^31^ In addition, musical taste relates to how we process music, particularly the one we prefer, for example, on a neural level,^40^ or on felt and perceived emotions.^41^ Unfortunately, musical taste research suffers from replication problems, which might partially be attributable to the different sets of genres selected for assessing taste.^13^, ^42^ Therefore, how to measure musical taste and which set of categories to select is of highest importance for a broad range of research questions related to music.

There have been different ways to address this question.^11^, ^14^ Studies investigating genre boundaries by applying both broad genre and narrow subgenres revealed systematic taste subgroups within genres, for example, mainstream and sophisticated subgenres,^17^, ^30^ or softer and harder ones, particularly shown for *rock*.^29^, ^30^, ^43^

Most studies relate musical taste to external criteria such as personality or socioeconomic factors. In our study, we focus on taste evaluations only. We compared evaluations of music liking from a broad set of 15 genre categories with 100 hierarchically nested, narrow subgenres, and two nonexisting subgenres (foil items). We added three representative artists to sharpen the definition and boundaries of subgenres.^12^, ^43^ Participants evaluated their liking of all the genres and subgenres or alternatively indicated that they did not know the item. We then applied a wide range of analyses, exploring how similar evaluations were between the two hierarchical levels of genre and subgenres. If evaluations were similar, the shorter list of genres might be more applicable in taste surveys than the long list of subgenres. Dissimilarities between genre and subgenre evaluations would necessitate a more differentiated assessment of musical taste.

### Outline of our methodological approach

We started our venture with analyzing responses to foil items and “don't know” responses (see section “Evaluation heuristics”). Our survey provides a hierarchical structure of subgenres nested in genres. In general, knowledge of hierarchical structures supports meaningful judgments on unfamiliar exemplars, shown in human classification learning,^44^ as well as applying machine learning to automatized classification labeling.^45^ We followed three objectives. First, evaluations of “knowing” and “liking” should be tightly linked.^46^ Liking of the foil items should be rather low, as they do not exist and, hence, should be not familiar. Similarly, genres with unfamiliar subgenres (many “don't know” responses) should be evaluated low. Second, given that evaluations under uncertainty follow heuristics that are based on statistical information,^47^ the evaluation of the foils should be related (e.g., correlated) to the evaluation of the other exemplars of this genre or the assigned genre category. Third, as genre categories are broad and include a range of exemplars on which evaluations can be applied, we expected lower frequencies for “don't know” responses for genre labels in comparison to subgenre labels.

We then turned to analyses comparing liking responses between genres and the existing nested subgenres. Coherence should result in matching responses. However, changing the category width also changes the construal level,^48^ which in turn can systematically influence attitudes and liking evaluations.^49^ In the section “Consistency of responses between genre and subgenres,” we show analysis of three aspects. First, we compared density functions of the evaluations. Second, we correlated responses between genres and nested subgenres for liking and familiarity (“don't know” responses). Third, we compared mean liking for different category widths (liking of genre vs. liking of nested subgenres). We then analyzed the homogeneity of liking subgenres from genres, exploring whether liking evaluations within each genre show systematic subgroups of taste.^17^, ^29^, ^30^, ^43^

To explore which subgenres people have in mind when thinking of genres, we developed a modeling approach to measure the typicality of exemplars. The analysis was based on models predicting liking of subgenres by their genre evaluation. In the final section, we report a second modeling approach to define the representative exemplars for liking of genre by models that predict liking of each genre based on the evaluations of all nested subgenres.

## MATERIALS AND METHODS

### Participants, sample size, ethics approval

Online data collection resulted in 804 completed surveys. Gender split almost binary (44.3% male, 53.4% female, 2.4% no answer), age ranged between 15 and 72 years (*M* = 27, *SD* = 10). Many participants reported a background in music (professional musicians: 21%; hobby musicians, who played an instrument/singing for more than 8 years: 36%; no instrument/singing: 19%).

Our sample was a convenience sample, matching what is prevalent in online musical taste research (e.g., young Western participants, interested in music). But subgenres of each genre should be known to a reasonable number of participants to enable collection of liking ratings. We therefore designed a quota assignment by the liking evaluations of the genres. Liking was defined by selecting a minimum of 4 on the 7‐point scale of liking from 1 = “not at all” to 7 = “very much,” which was a rather liberal criterion. We aimed for 15 (genres/quota groups) × 50 (participants) = 750 (total number) and stopped data collection, when the quota assignment ranged between 49 and 55 participants for all 15 genres. At this point, none of the volunteers were excluded from participation. The liberal criterion did not result in a proportional distribution of favorite music (as defined by high liking).

Before starting the survey, participants were informed about the content and scientific background of the study as well as the voluntary and anonymous data collection. Participants gave consent to participate by proceeding the questionnaire. All materials were in the German language. Data were collected according to the principles of the Declaration of Helsinki. All procedures were approved by the Ethics Council of the Max Planck Society.

### Musical taste questionnaire

As a first step, we defined the list of genres. For this, we compiled genre labels from a total of 20 record shops, streaming services, music magazines, and webpages (e.g., Saturn, last.fm, etc.) and determined the genres that appeared in at least half of the 20 sources, which were *alternative*, *blues*, *classical*, *country*, *electronica*, *folk*, *hip‐hop/rap*, *jazz*, *metal*, *pop*, *reggae*, *rock*, *soul*. We added *techno*, *house*, and *volkstümliche Musik* (*volkst*.) because of their popularity (based on evaluations published by statista [https://de.statista.com] and the German music industry [BVMI, https://www.musikindustrie.de]) and excluded *alternative* as being not distinct enough. Note that there is no universal English translation for traditional German music, so we will keep the German expression *volkstümlich* (*volkst*.) for this genre for the rest of this manuscript. We then asked music producers, musicians, and musicologists with expertise in popular music studies to assemble hierarchically nested subgenres that represent the width of those 15 higher level genres as best as possible. The task was to assemble “as many as necessary, as little as possible.” In addition, they should name up to three representative musicians for each subgenre. We collected responses by questionnaires and in focus groups. In new focus groups, we refined the final selection of subgenres and assigned musicians, for example, *soul* included *classic RnB* (e.g., Aretha Franklin, etc.), *soul‐funk* (e.g., James Brown, etc.), *60ies soul* (e.g., The Temptations, etc.), *70ies soul* (e.g., Curtis Mayfield), *pop RnB* (e.g., Alicia Keys, etc.), and *neo‐soul* (e.g., D'Angelo, etc.; see Table S1 for a complete list of the genres, subgenres, and artists). We added two nonexisting subgenres and bands (foil items): *purega rap* (starry‐eyed rap) with the artists Hoodhero, HeavG, and Meek Divine, and *poolem metal* with the artists New Clank, non se ipse, and Drop Dead Disturbed. The foil items and related artists did not exist, but were presented as respective subgenre of the genres *rap* or *metal*. Names of the foils and artists were created to represent imaginary subgenres that would eventually fit into the overall appeal of the related genre. All genres and subgenres were evaluated on 7‐point scales of liking from 1 = “not at all” to 7 = “very much,” or alternatively could be marked as unfamiliar (““don't know” this item”).

#### Procedure

The questionnaire was set up and provided to the participants using the survey tool www.unipark.de. The serial order of items in the online questionnaire was fixed: Evaluations of all genres came first and were presented on one page. On several consecutive pages, the nested subgenre belonging to one genre were presented, each list of subgenres on one page. Evaluations of the nested subgenres started with the question: “How much do you like music from the following subgenres of [corresponding genre label inserted]?” The evaluation part was followed by further questionnaires, and the assessment of demographic information. The serial order of responses on each page could be chosen freely.

#### Data treatment and analyses

Our study was exploratory, and in most cases we did not favor the null‐ or alternative hypothesis (i.e., when asking about homogeneity, typicality, representativeness). However, a priori calculations of sample sizes (including Bonferroni corrections for multiple testing) indicated that our sample size should be sufficient (calculations by G‐power,^50^ correlations: *N* = 398; linear regressions, *N* = 269; recommendation^51^ for factor analyses: *N* = 500). Given the exploratory approach, we report effect sizes when comparing means. Note that we report two different ways to create means: collapsing observations across ratings (means for participants) or across participants (i.e., means for genres, *N* = 15).

To explore the homogeneity of liking subgenres from genres, we calculated 15 factor analyses, one for all nested subgenres related to one genre. As potential factors were assumed to correlate, we decided on oblique rotation. We applied the function *parallel()* from the *nFactors* package in R to decide on the number of factors.^52^ Note that we decided for a factor analyses and not principal components analyses (PCA), because we assume—if anything—underlying latent variables driving potential groups of subgenres.

The analysis of the typicality of exemplars was based on 100 linear models of genre ratings predicting nested subgenre ratings, one regression model for each subgenre (e.g., liking of *blues* predicting liking of subgenres nested within *blues*: *blue*_1_, *blue*_2_, *blue*_3_, *blue*_4_, *blue*_5_, *blue*_6_). We interpreted the explained variances (*r* ^2^) of each nested subgenre as a measure of the typicality of exemplars. We calculated thresholds for defining “rather typical” and “rather atypical” by the mean *r* ^2^ across models of subgenres from one genre ± their standard deviation (*SD*). As a control, we also calculated explained variance of the linear models of all other genre ratings predicting the same set of subgenres as before (e.g., liking of *reggae, hip‐hop/rap*, and so forth, predicting liking of subgenres nested within *blues*). We calculated mean (*M*), *SD*, and median across the *r* ^2^ for those models belonging to the same substyles (e.g., from *blues*), which can be interpreted as estimates of chance for the reported typicality measures.

In the section “Representative exemplars for liking of genre”, we ask which subgenres together correspond to the genre rating by taking the liking of the nested subgenres as predictors for liking the genre. Because of correlated ratings across subgenres, we applied random forest models and calculated the “relative importance” of each predictor. We determined the most important predictors for the most parsimonious model based on the variables’ relative importance means and standard deviations, as well as out‐of‐bag (OOB) error rates.

## RESULTS

### Evaluation heuristics

#### Foil items and “don't know” responses

**FIGURE 1:**
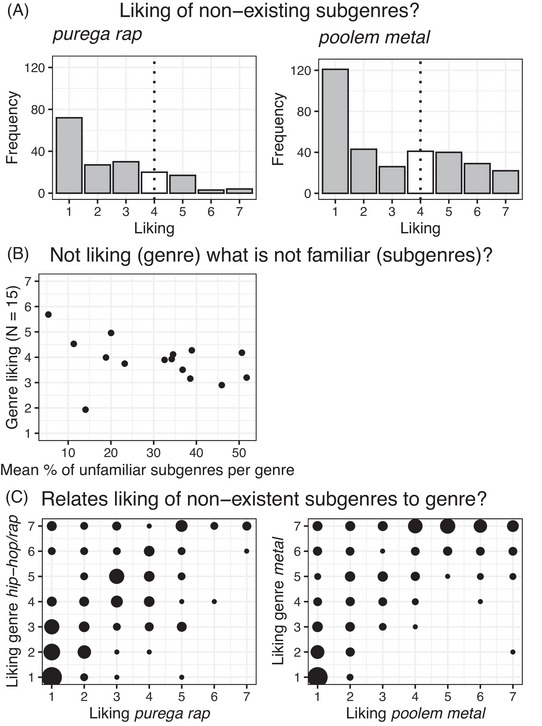
Liking and unfamiliarity. (A) Frequencies of liking evaluations (7‐point scale) for the two nonexisting subgenres. When subgenres were evaluated, they were mostly not liked. (B) Percentage of unfamiliar subgenres for each genre related to the liking of their genres. There is a tendency of a negative correlation. (C) Liking evaluations of the nonexisting subgenres and their genres are correlated. As individual data overlap, the size of the points denotes the frequency of cases.

The foil items were detected (i.e., answering “don't know”) by the majority of participants (*hip‐hop/rap*: 79%; *metal*: 60%), but a considerable number of participants evaluated the foils (*hip‐hop/rap*: *n* = 173; *metal*: *n* = 322). Their liking responses were analyzed further. First, we compared frequencies of observed low and high liking (midpoint 4; Figure 1A) with what can be expected by chance (50% low liking responses, 50% high liking responses). Since the foils did not exist, their familiarity should be low and, accordingly, liking evaluations should also be low. Both *χ* ^2^ goodness of fit tests were significant, for *hip‐hop/rap*, *χ* ^2^(1, *n* = 173) = 72, *p* < 0.001, and *metal*, *χ* ^2^(1, *n* = 322) = 35, *p* < 0.001. There were more observations on the low liking part of the scale than the high liking part. We then related the unfamiliarity of existing subgenres (percentage of “don't know” responses) with mean liking of the genres (*N* = 15). The correlation showed a tendency, *rho*(13) = −0.37, *p* = 0.173, BF_01_ = 1.08 (Figure 1B).^53^ Hence, there is some indication for “not liking what is not familiar.”

Second, we correlated subjects’ liking of the foil items with liking of the associated genre (*hip‐hop/rap*: *rho*(171) = 0.63, *p* < 0.001, *metal: rho*(318) = 0.75, *p* < 0.001; Figure 1C), and likewise with mean liking of existing subgenres, M(sub) (*hip‐hop/rap: rho*(171) = 0.76, *p* < 0.001, *metal: rho*(320) = 0.87, *p* < 0.001). To control for chance and test for an overall similarity between evaluations regardless of genre content, we correlated also the foil of *hip‐hop/rap* with the evaluations of *metal* and vice versa. None of these correlations were significant and all correlation coefficients were below *rho* < 0.10, all BF_01_ ≥ 4.55. That is, evaluations of the foil items were based on knowledge about the associated genre and related subgenres.

Third, the frequency of “don't know” answers differed between genre and subgenre categories as predicted, *t*(14) = 7.86, *p* < 0.001, *d* = 2.03, 95% CI [1.12, 2.92]. For some genres, the difference was quite large (Table 1). For example, for *blues* the genre was highly familiar (98% liking evaluations, unfamiliar to 2% participants only), but the subgenres of *blues* were rather unfamiliar (33%–68% “don't know” responses, *M* = 51%; Figure S1 shows the percentage of “don't know” responses of each subgenre). That is, broad genre categories have higher chances to be evaluated than narrow subgenres categories.

**TABLE 1:** Liking and familiarity of genres and mean across subgenres.

	Genres		Subgenres			
	Mean liking (*SD*)	% “don't know”	Mean M(sub)	% “don't know” responses across subgenres		
				Mean (*SD*)	Lowest	Highest
*Blues*	4.18 (1.68)	1.7	4.37 (1.35)	50.66 (13.62)	32.46	67.79
*Classical*	4.96 (1.71)	0.4	4.25 (1.36)	20.04 (15.89)	2.11	46.89
*Country*	3.16 (1.57)	0.8	3.67 (1.42)	38.56 (20.31)	15.55	57.21
*Electronica*	3.51 (2.02)	17.9	3.36 (1.36)	36.78 (18.50)	12.69	60.07
*Folk*	4.11 (1.79)	3.6	4.40 (1.55)	34.54 (7.91)	25.50	40.17
*Hip‐hop/Rap*	3.75 (1.87)	0.3	3.45 (1.45)	23.22 (17.45)	3.86	53.98
*House*	3.20 (1.92)	2.6	3.23 (1.69)	51.74 (10.90)	34.08	65.05
*Jazz*	4.27 (1.81)	0.4	4.08 (1.44)	38.93 (15.54)	18.91	63.56
*Metal*	3.99 (2.29)	0.4	3.56 (1.57)	18.83 (12.38)	3.61	38.81
*Pop*	4.53 (1.71)	0.1	3.49 (1.08)	11.31 (10.66	0.50	31.22
*Reggae*	3.93 (1.64)	0.9	4.13 (1.48)	34.20 (19.10)	7.59	52.49
*Rock*	5.68 (1.45)	0.5	4.41 (1.12)	5.39 (6.29)	0.25	19.78
*Soul*	3.90 (1.73)	2.0	4.22 (1.47)	32.56 (17.51)	5.10	55.22
*Techno*	2.90 (1.92)	0.4	2.53 (1.65)	45.96 (11.05)	35.70	58.96
*Volkst*.	1.93 (1.33)	0.1	2.16 (1.15)	14.04 (11.66)	4.60	32.59

We want to add one aspect of the data here: Overall, 377 participants responded with liking evaluations for one or both foils, whereas 427 responded “don't know” for both foils. As “don't know” is the correct response to a nonexisting subgenre, these participants might be regarded as more reliable. However, we noted that participants of this subset (*n* = 427) consistently responded “don't know” to subgenres more often than the other subset (*n* = 377; see Figure S2). One interpretation is that participants differed by their response criterion to respond to more unfamiliar subgenres: Some participants decided answering “don't know”, others based their response on associations of the label and follow heuristics. When the threshold to answer “don't know” is low, chances are high that foils are correctly rejected. Responses on foils, then, do not necessarily say much about the reliability of the participants.

### Consistency of responses between genre and subgenres

#### Response distributions

**FIGURE 2:**
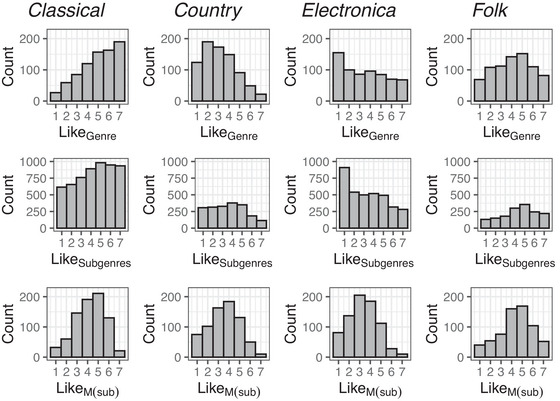
Example distributions of liking ratings (7‐point scale) of four genres. First row: histograms for the broad genre category. Second row: histograms for the narrow subgenre categories. Note, subplots depict raw data, not weighted for the number of responses or number of subgenres for the genre categories. Third row: histograms for the mean across subgenres, M(sub).

Figure 2 depicts histograms of the liking ratings on the level of genres, subgenres, and the mean across subgenres assigned to each genre, M(sub). Note that the first two rows depict raw data, which were further processed (i.e., averaged) for most analyses. We selected four examples that visualize differences and similarities: *classical*, *country*, *electronica*, and *folk*. The ratings of genres were arbitrarily distributed (Figure 2, first row): The genre *classical* was left‐skewed, *country* was right‐skewed, *electronica* was almost equally distributed with an exceptional peak on the lower end, *folk* had an almost symmetrical unimodal distribution. Interestingly, when taking all ratings for the subgenres into account (not weighted for the number of given answers or subgenres), distributions became shallower (Figure 2, second row), and the distributions showed no longer a steep upward (*classical*) or downward (*country*) slope. But for *electronica* the exceptional peak for liking = 1 remained. In other words, the standard deviation of the distribution increased for distributions on subgenre responses in comparison to genre responses, *t*(14) = 2.50, *p* = 0.025, *d* = 0.65, 95% CI [0.08 1.20], suggesting more differentiated responses when asking for liking of subgenres than genres. However, the confidence interval for Cohens *d* indicates a nonreliable effect. The four example distributions of M(sub) (Figure 2, third row) were more similar for mathematical reasons: distributions of means become more normal (central limit theorem).

Despite the differences between the distributions of the genre ratings and M(sub), both measures were highly correlated, ranging between *rho*(798) = 0.49 for *rock* and *rho*(787) = 0.82 for *metal*, both *p*’s < 0.001 (see Table S2 for a complete list of correlation coefficients; see Figure S3, depicting distributions of subgroups of participants based on their genre rating).

#### Correlations on genre level

We repeated the reported correlation between liking evaluations of genres and M(sub), averaging for genres (*N* = 15), and not participants. The result was significant as well, *rho*(13) = 0.76, *p* = 0.001. The Spearman's rank correlation of unfamiliarity (“don't know” responses) between genres and the mean across subgenres was significant, too, *rho*(13) = 0.54, *p* = 0.04, showing that the ranking from unfamiliar to familiar genre and subgenres was related, despite the strong difference in numbers (familiar genre but unfamiliar subgenres). In both cases (liking/unfamiliarity), correlations were significant but not perfect (*r* = 1), that is, there were differences in exact rank orders (see Figure S4). We report factor analyses of genre evaluations in the Supporting Information section “Distinctiveness of taste dimensions: clarity of genre boundaries.” The resulting taste dimensions differed depending on the selected measure, genre liking or M(sub), but were more plausible for the genre ratings.

#### Category width and liking evaluations

Comparisons of liking evaluations between genre and M(sub) did not indicate a systematic bias: Nine comparisons showed higher liking for genre than M(sub) (Cohen's *d* for *classical*: 0.65, *electronica*: 0.13, *hip‐hop/rap*: 0.30, *house*: 0.15, *jazz*: 0.24, *metal*: 0.34, *pop*: 0.76, *rock*: 1.04, *techno*: 0.43), four the opposite (*country*: 0.34, *reggae*: 0.12, *soul*: 0.21, *volkst*.: 0.23), and two were so small that we avoid an interpretation of the direction of the effect (*blues: 0.02*, *folk: 0.05*; Table 1 lists liking of genres and mean liking of nested subgenres; Table S2 reports *t*‐tests). It is notable that the biggest difference in liking evaluations and the largest effect sizes were shown for the two popular musical genres *pop* and *rock*: 1.05 and 1.29 units on the 7‐point scale, respectively. Importantly, there was no systematic bias of category width on taste ratings. That is, differences were not consistently pointing into one direction.

### Homogeneity of liking subgenres from genres

**FIGURE 3:**
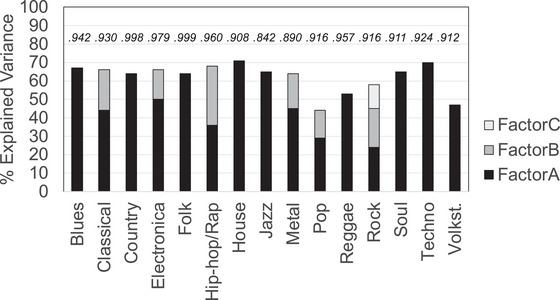
Results of 15 different factor analyses, one for each genre, including nested subgenres. For most genres, liking evaluations loaded on one factor (A), indicating a coherent dimension of musical taste. Others loaded on two (A, B) or three factors (A–C). The *y*‐axis depicts explained variance of the factor(s). The values on top show the CFI for each factor analysis.

Detailed results of the 15 factor analyses are reported in Tables S3–S5. Figure 3 depicts the main results: Nine genres consisted of one factor (Factor A), indicating that responses for liking were coherent across all subgenres (*blues*, *country*, *folk, house*, *jazz*, *reggae*, *soul*, *techno*, *volkst*.). The other genres split into two (Factors A and B) or three meaningful factors (Factors A–C). The subgroups of two and three factor solutions were interpretable. *Classical* split into two factors, one for the recent developments (e.g*., class*_8_ = *classical modern music*, *class*_9_ = *avant‐garde*), and another for music from the medieval ages to the early 20th century. For *electronica*, the more popular and dance‐oriented subgenres (*elec*_6_ = *Eurodance*, *elec*_7_ = *EDM*) loaded on one factor, all others on the second. *Hip‐hop/rap* split roughly into *hip‐hop* (*hip*_1_–*hip*_4_ plus hip_7_ = *alternative rap*) and *rap* (hip_5, 6, 8_). For *metal*, almost all subgenres loaded together on one factor but the subgenres popular in Germany (*metal*_8_ = *alternative/nu metal*, *metal*_11_ = *industrial/Neue Deutsche Härte*). In *pop*, the three subgenres including singer–songwriters of English (*pop*_2_), German (*pop*_3_), or French origin (*pop*_4_) singled out on one factor, all other loaded together. Finally, *rock* split into three factors, one for *German rock* (*rock*_1_ = *Deutschrock*, *rock*_2_ = *Neue Deutsche Welle [Engl. New German Wave]*), another for the subgenres of the *roots of rock* (*rock*_3_ = *rock'n’roll*, *rock*_4_ = *rockabilly*, *rock*_5_ = *beat*), lastly for the more typical *classic rock* (*rock*_6_ = *psychedelic rock/progressive rock*, *rock*_7_ = *hard rock*, *rock*_8_ = *punk rock*, *rock*_9_ = *alternative (post‐punk, wave, gothic*).

Most analyses’ solutions were satisfactorily (see comparative fit indices, CFIs, in Figure 3, and Tables S3–S5), that is, our genre categories were nicely defined by one or more coherent sets of subgenres. However, summed variance was low (<50%) for a few genres, for example, *pop* and *volkst*., indicating less coherent tastes for those genres.

Note that the number of subgenres differed based on the experts’ opinion, and ranged between three and ten. As factor analyses are tools for data reduction, this method is less plausible for data sets with low numbers of items. In fact, given the advice to accept factors with a minimum of three items,^54^ results for genres with less than six subgenres (to result in at least two factors) have to be interpreted with caution (e.g., *folk*, *reggae*, *country*, *techno*). We included these genres in the analyses, because factor loadings are still informative as well as model fits, which were similarly high between subgenres (loadings) and similarly sufficient (fits).

### Typicality of exemplars

**FIGURE 4:**
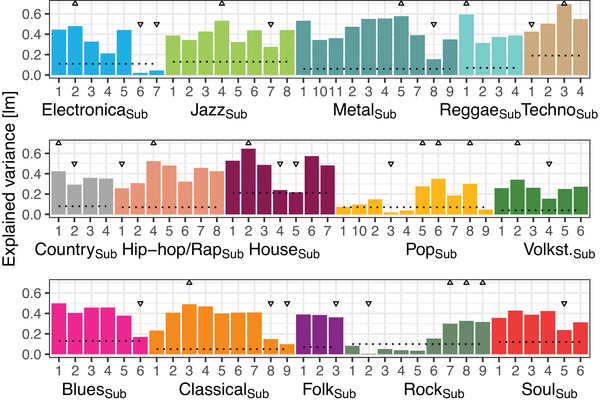
Model results of 100 linear models (lm) of genre liking predicting the nested subgenres. The explained variance (*r* ^2^) of each model is depicted. Digits on the *x*‐axis represent the names of subgenre by its number (see Table S1 for a complete list of subgenres and related artists). Triangles up symbolize typical subgenre exemplars of the genre, triangles down atypical ones. For the names of the typical and atypical exemplars, see the text. The dotted lines depict the control measure, see the text.

Explained variances (*r* ^2^) of the 100 linear models are depicted in Figure 4, ranging from *r* ^2^ = 0.005 (*rock*_2_ = *Neue Deutsche Welle*) to *r* ^2^ = 0.700 (*techno*_2_ = *Detroit techno*). For some genres, explained variance was overall rather weak to moderate (*pop* or *rock*, mean *r* ^2^ across subgenres ≤ 0.16), for others substantial (*metal, techno, house*, mean *r* ^2^ across subgenres ≥ 0.42).^55^ The triangles in Figure 4 mark the more or less typical subgenres (pointing upward or downward respectively). For most genres, one most typical subgenre was defined (e.g., *jazz*_4_: *cool jazz* (Miles Davis Capitol Orchestra); *reggae*_1_: *roots reggae* (e.g., Bob Marley); *classical*_3_: *classical* (e.g., symphonies and string quartets from Mozart). The popular genres *pop* and *rock* had more than one typical subgenre. For *blues*, *folk*, and *soul*, no subgenre stood out as typical. We also defined less typical subgenres (e.g., *jazz*_7_: *smooth jazz* [e.g., Norah Jones], or *metal*_8_: *alternative/nu metal* [e.g., Limp Bizkit]).

We calculated several measures to control for chance by exchanging the predictor for the same type of linear models. Figure 4 shows *M* + 1 *SD* of all model outcomes (*r* ^2^) belonging to the same set of subgenres (depicted on the *x*‐axis) as dotted line (with predictors were liking evaluations from the unrelated genres). As the model outcomes were exponentially distributed, *M* and *SD* are strongly affected by outliers (i.e., rare but high values of *r* ^2^), and the median is arguably a more appropriate measure of control but less conservative. It is notable that all medians across models were rather low, ≤0.044 (see Table S6 for a complete list of measures), indicating that most subgenres’ likings were predicted more likely by the related than the unrelated genres. For *pop* and *rock*, model outcomes for several subgenres did not exceed the *M* + 1 *SD* threshold of the control measure (dotted line in Figure 4), showing a rather loose relation between genre and nested subgenres. For some sets of subgenres, *M* + 1 *SD* were rather high, for example, for *house*_sub_ and *techno*_sub_. The underlying reason was high cross‐correlations between evaluations of genre and subgenres from *house*, *techno*, and *electronica*. For example, liking of *techno* predicted *hous*_1_, *hous*_2_, *hous*_6_, *hous*_7_ with *r* ^2^ ≥ 0.46. The slightly elevated dotted line for *jazz* was based on a few cross‐correlations between liking the genre *jazz* and subgenres from *blues*, *reggae*, and *soul*.

### Representative exemplars for liking of genre

**FIGURE 5:**
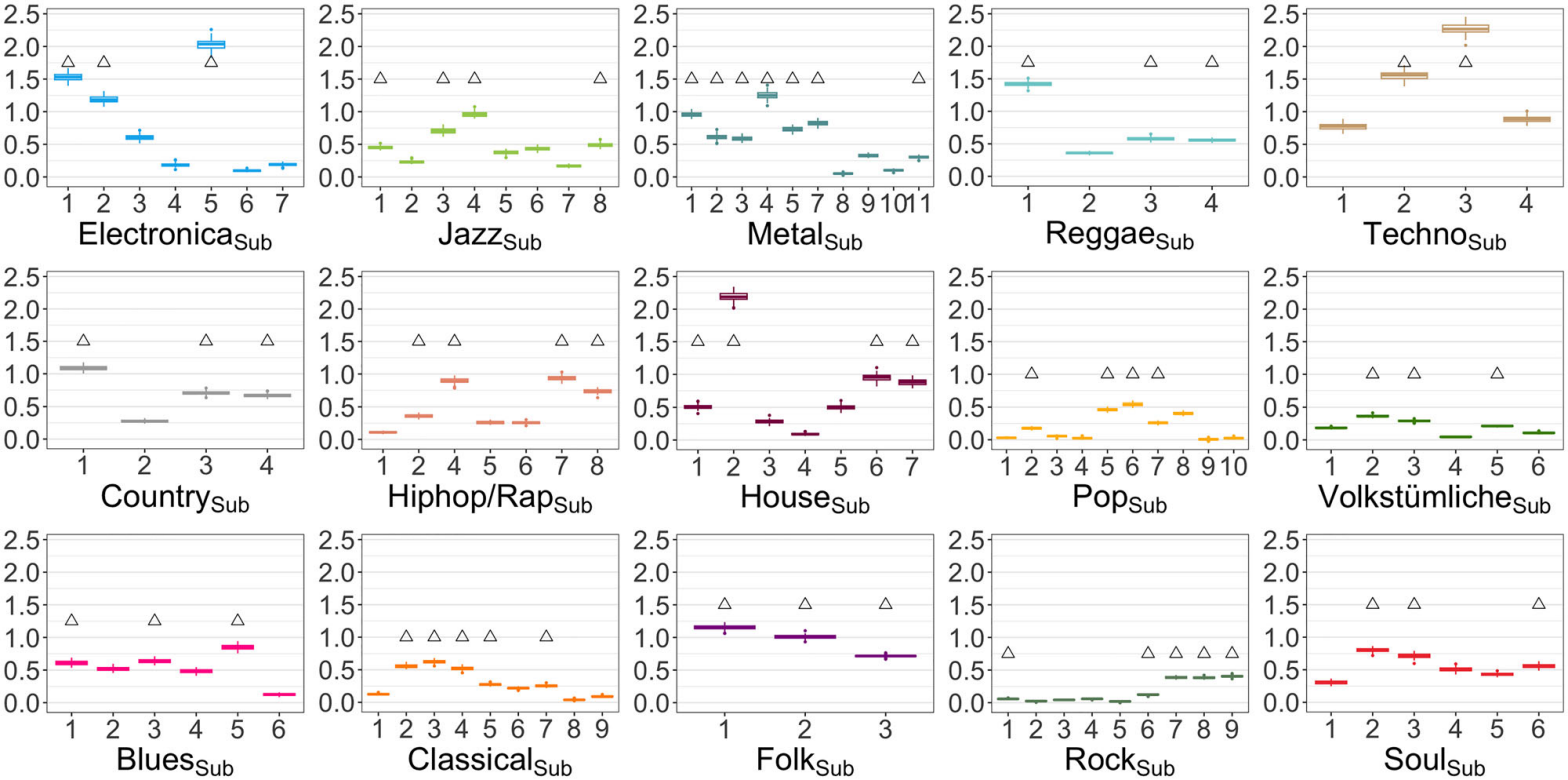
Model results of 100 random forest models. The *y*‐axis depicts the mean relative importance across the 100 repetitions of the model estimates for the nested subgenres predicting the genre rating, when all related subgenres are included in the model at the same time. Digits on the *x*‐axis represent the names of subgenre by its number (see Table S1). The best predictors of the most parsimonious model are marked by triangles (selection by the *vsurf* package in *R*).^50^

Figure 5 depicts the relative importance of all subgenres’ liking to predict liking of the associated genre. We selected the most important predictors (marked by an upward triangle) by the most conservative criteria applying the *R* package *vsurf* ^56^ (for full results see Table S7). We defined those subgenres as being most representative for the genre. Interestingly, for all genres, the majority of subgenres contributed to the model fit, and not many could be excluded without reducing the goodness of fit. Indeed, when using the most liberal criteria for subgenre selection (thresholding step), all nested subgenres showed importance for predicting the related genre (with one exception: *pop*_9_ for *pop*). That is, all subgenres’ evaluations contributed to the variance of liking the associated genre in this model approach. When interpreting Figure 5, this needs to be taken into account, because the marked selections of subgenres are just one of several possible interpretations.

## DISCUSSION

The current study explored category width in the arts domain by exploring liking evaluations for musical genre and subgenres. The first important observation was that a high percentage of participants evaluated subgenres and musicians that do not exist (20%–40%). On the one hand, evaluations of these unfamiliar items resulted in low liking, as expected by the link between familiarity and liking.^46^ On the other hand, evaluations were highly related to existing subgenres of the same genre or the superordinate genre category. Hence, related familiar items seem to work as placeholders for evaluations under uncertainty (e.g., foil items). Heuristics also come into play for existing subgenres and genres. For example, people evaluate genres without knowing the subgenres (e.g., *blues*). The rank orders (Spearman correlations) of liking or of “don't know” responses were highly related between the genre and the subgenre categories. These are important findings as they contradict the argument that evaluations on the level of genre categories and subgenres categories would be not comparable.^16^, ^17^

Even though there was high consistency between liking music assessed by subgenres and assigned genres, we also revealed notable differences. Liking evaluations differed by category level (broad genres vs. narrow subgenres), but no systematic bias of width occurred (as predicted by research into consumer preference^43^): some genres were more liked than the nested subgenres, some less. This does not rule out that an effect of width interacted with being a fan of that genre, which has been shown in the context of political ideologies.^57^ The density functions of responses revealed differences: The standard deviations of nested subgenres had a tendency to be wider than the genre evaluations (as predicted by construal theory).^49^ The distributions of subgenre means were unimodal (central limit theorem), whereas evaluations of genres or subgenres can have any arbitrary distribution. This has consequences for statistical analyses (e.g., when normally distributed variables are required). If a researcher is more interested in the extreme values at the edge of a range, working with simple frequencies and not means is sensible.

The factor analyses revealed that evaluations of subgenres belonging to the same genre were highly similar in nine cases for which the genre labels are suitable categories.^1^ For the two or three factor solutions, subgenres were not differentiated into the softer/harder or mainstream/sophisticated distinction, thus failing to replicate earlier studies. However, they match other approaches^58^ in genre theory: distinct tastes for avant‐garde subgenres (i.e., *classical modern* and *avant‐garde* music differentiates from other *classical* music), scene influences (e.g., *nu‐metal* and *industrial* differentiates from other *metal* music, *EDM* splits from *electronica*, etc.), or traditional subgenres (i.e., *rock'n’roll* and *rockabilly* differentiates from other *rock* music). Based on the resulting 22 factors and along with a few genres that exhibited low loadings on their respective factors and have a distinct listenership (e.g., *Schlager*), we suggest a selection of 24 genre labels to assess musical taste in Germany—adaptable for other Western countries as well: *blues*, *classical music*, *classical modern and avant‐garde music*, *country*, *electronica*, *Eurodance and EDM*, *folk*, *hip‐hop*, *rap*, *house*, *jazz*, *metal*, *nu‐metal and industrial*, *pop*, *singer–songwriter and chanson*, *soundtrack*, *reggae*, *rock'n’roll and rockabilly*, *rock*, *Deutschrock and NDW*, *soul*, *techno*, *Schlager*, and *volkstümliche Musik*.

Furthermore, we identified typical and atypical subgenres (Figure 4), and the most representative subgenres for each genre (Figure 5). Together, our results will guide researchers interested in musical taste of specific subgenres, and whether those are factored in the evaluation of the associated genre. In addition, results showed that application of the genres‐labels *pop* and *rock* is particularly problematic: the explained variances of linear models were low in comparison to the other genres (Figure 4), *pop* resulted in the smallest overall explained variance in the factor analyses (Figure 3), *rock* resulted in a three‐factor solution, and for *pop* and *rock* the liking evaluations strongly differed between evaluations on the genre and the subgenres level (see section “Category width and liking evaluations”).

Assessing taste by broad genres or narrow subgenres—where do we stand? We started this investigation with a strong negative bias. In musical taste research, a common critique is that broad genre categories are not meaningful to assess taste sufficiently.^15^, ^16^ Findings from our lab strongly support this.^17^ Given this negative bias, we were surprised by the high consistency between liking evaluations of genres and related subgenres. Therefore, we argue that for many applications, assessing musical taste by a limited set of broad genre categories is sufficient. Statements such as musical preferences were “genre‐free,”^59^ are at odds with our results. However, we applaud research on music listening behavior avoiding the concept of genre,^60^ or approaches stressing the fact that genres have limited explanatory power,^15^, ^59^ or other factors constitute preference factors.^61^, ^62^, ^63^ Also, we agree that taste is more differentiated than a list of broad genre categories can assess.^16^, ^17^ For example, in our study, the demonstrated correlations were not perfect (*r* = 1), neither were model weights in regressions, and rank orders were related but not exactly the same (see Figure S4). Substitution, then, is not perfect but prone to errors, which can be more or less important in different research contexts.

Finally, we want to point out that evaluations in the arts might focus more on differences than similarities between elements of categories. In the arts, we rather ask what makes a specific artwork special, and not what are the similarities between elements of a genre, or between subgenres, and genres, whereas in everyday life we treat similar objects equally.^1^ More research is needed to understand whether basic categorization principles apply to the arts in general, as indicated by our findings.

## AUTHOR CONTRIBUTIONS

**Elke B. Lange**: Conceptualization; methodology; formal analysis; resources; data curation; writing—original; writing—review and editing; visualization; supervision; project administration. **Emily Gernandt and Julia Merrill**: Methodology; writing—review and editing.

## CONFLICT OF INTEREST STATEMENT

The authors declare no conflicts of interest.

## PEER REVIEW

The peer review history for this article is available at: https://publons.com/publon/10.1111/nyas.15404.

## Supporting information




**Supporting Information**.

**Supporting Information**.

**Supporting Information**.

**Supporting Information**.

**Supporting Information**.

**Supporting Information**.

## Data Availability

The items of the survey, all primary data, and all analysis scripts are available at https://osf.io/4nz93
