# Supplementary figures and images for: Categorizing music by genres
Source: Ann N Y Acad Sci. 2025 Jul 9;1550(1):227–38. doi: 10.1111/nyas.15404 (PMC12412717; doi:10.1111/nyas.15404)

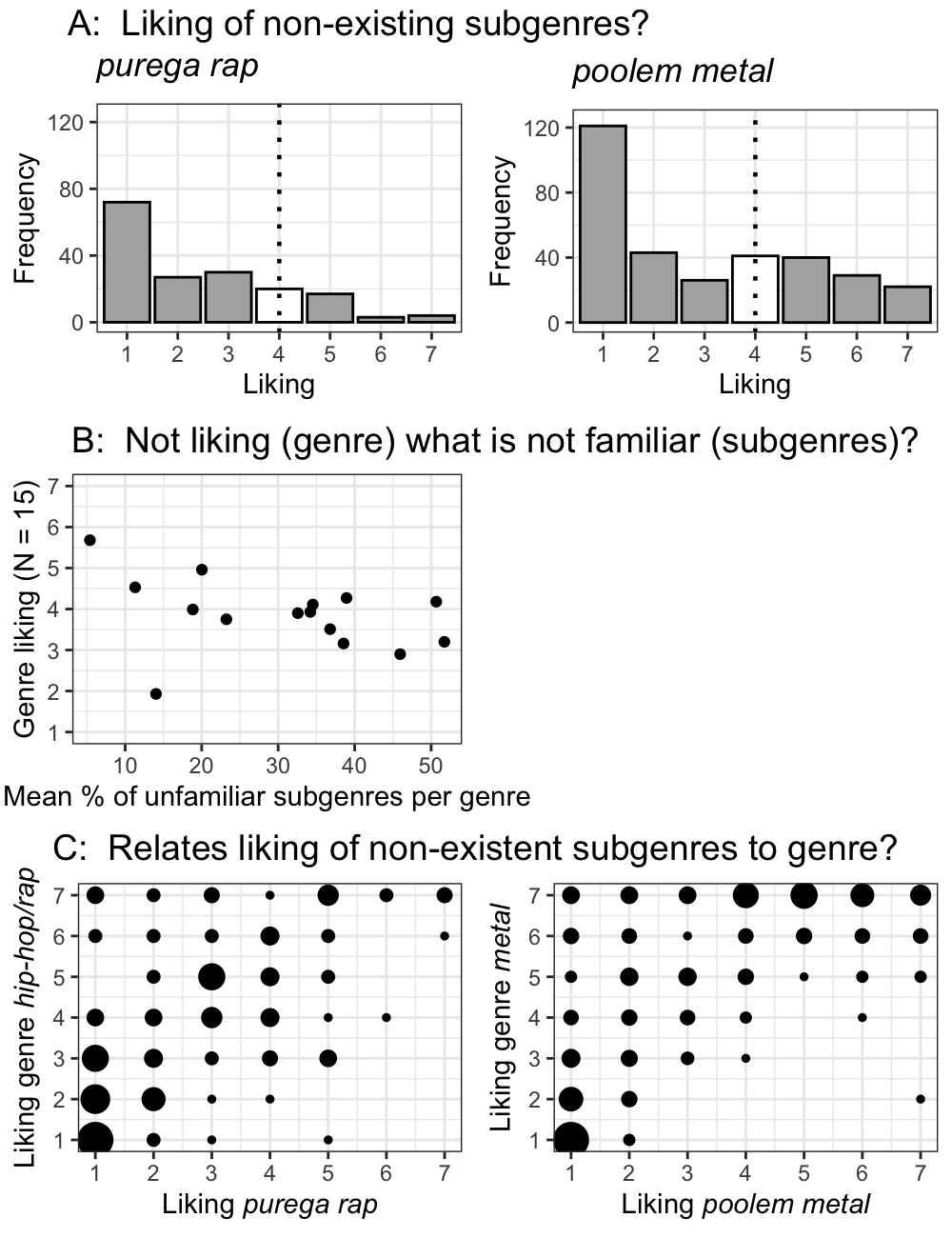

Supplement: Supplementary file 1 — Supporting Information. [file NYAS-1550-227-s006.png]

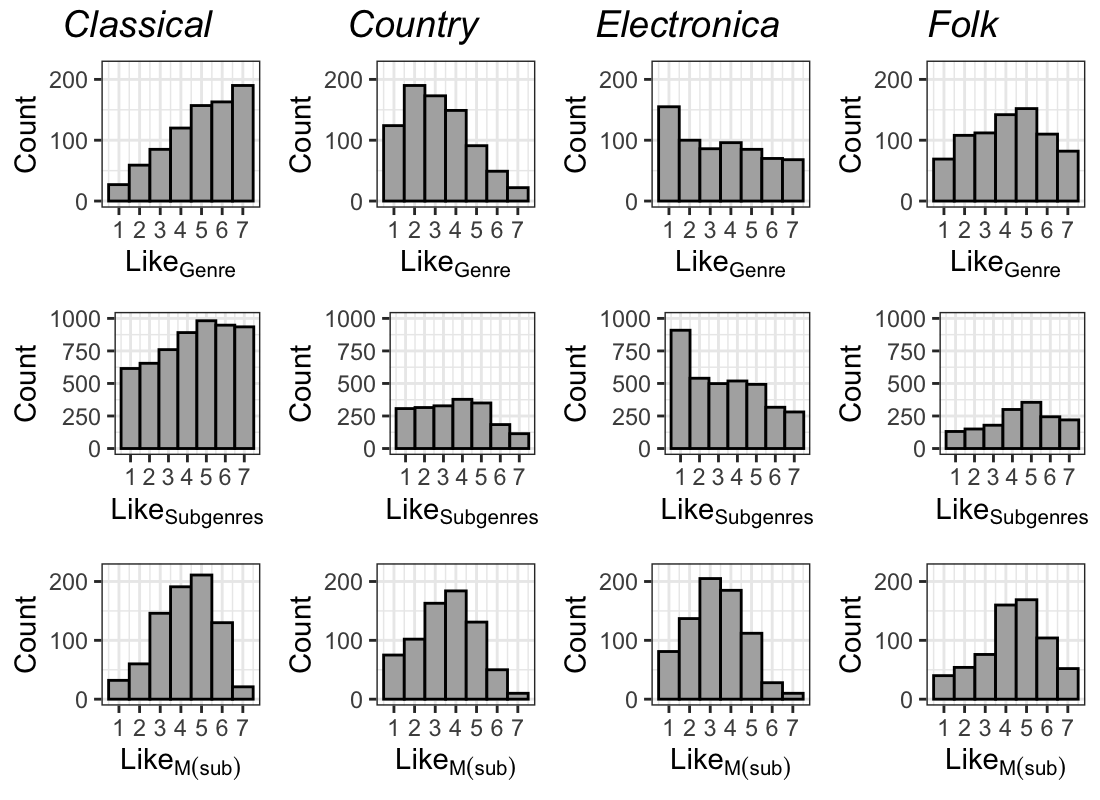

Supplement: Supplementary file 2 — Supporting Information. [file NYAS-1550-227-s005.png]

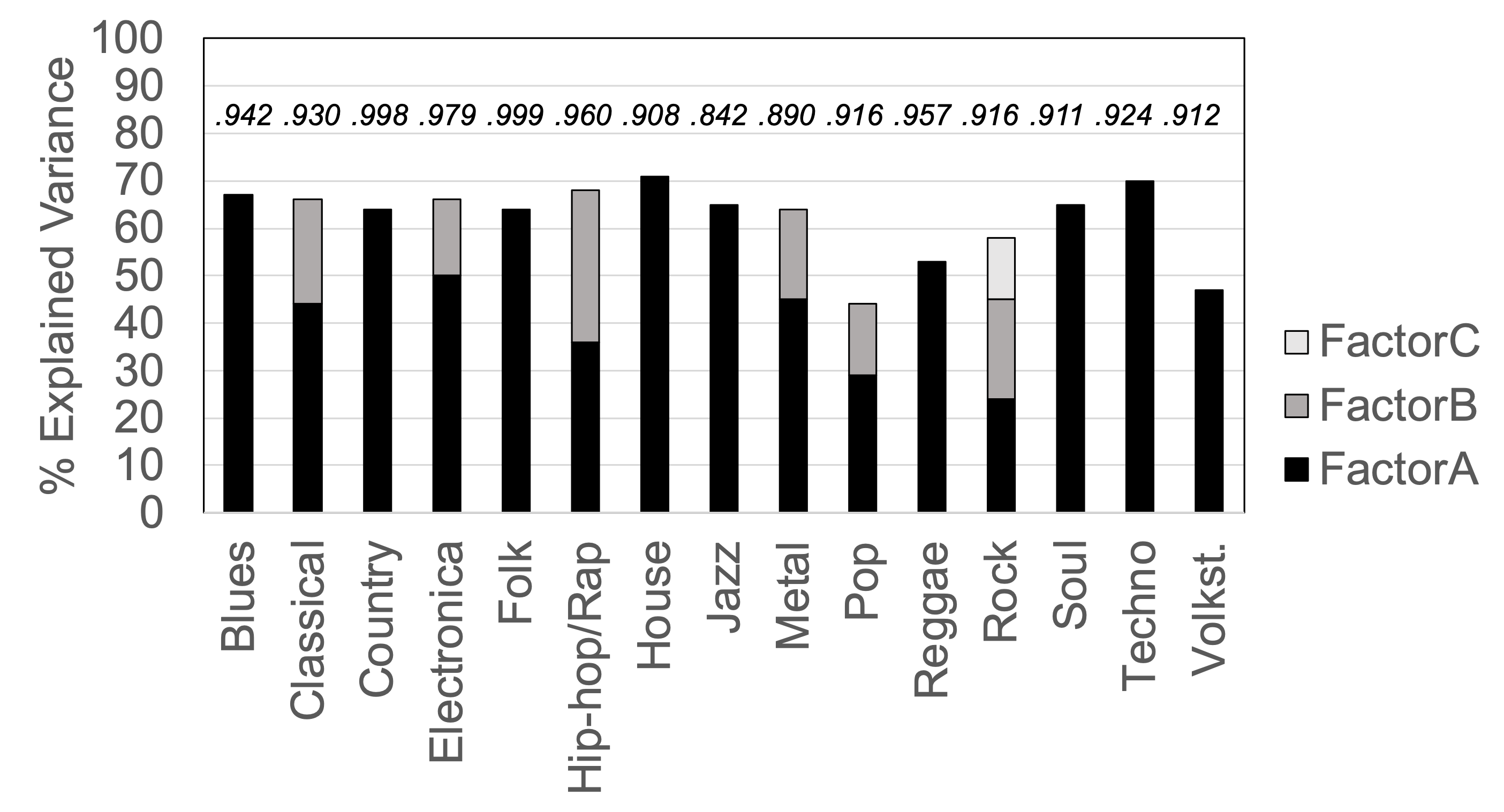

Supplement: Supplementary file 3 — Supporting Information. [file NYAS-1550-227-s002.png]

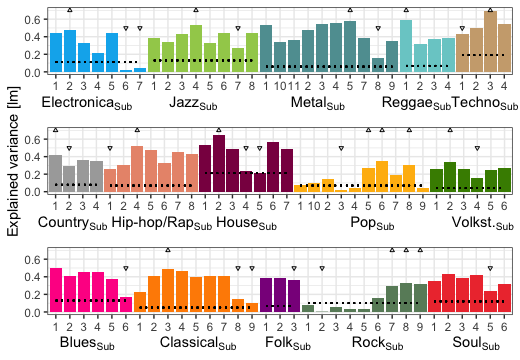

Supplement: Supplementary file 4 — Supporting Information. [file NYAS-1550-227-s003.png]

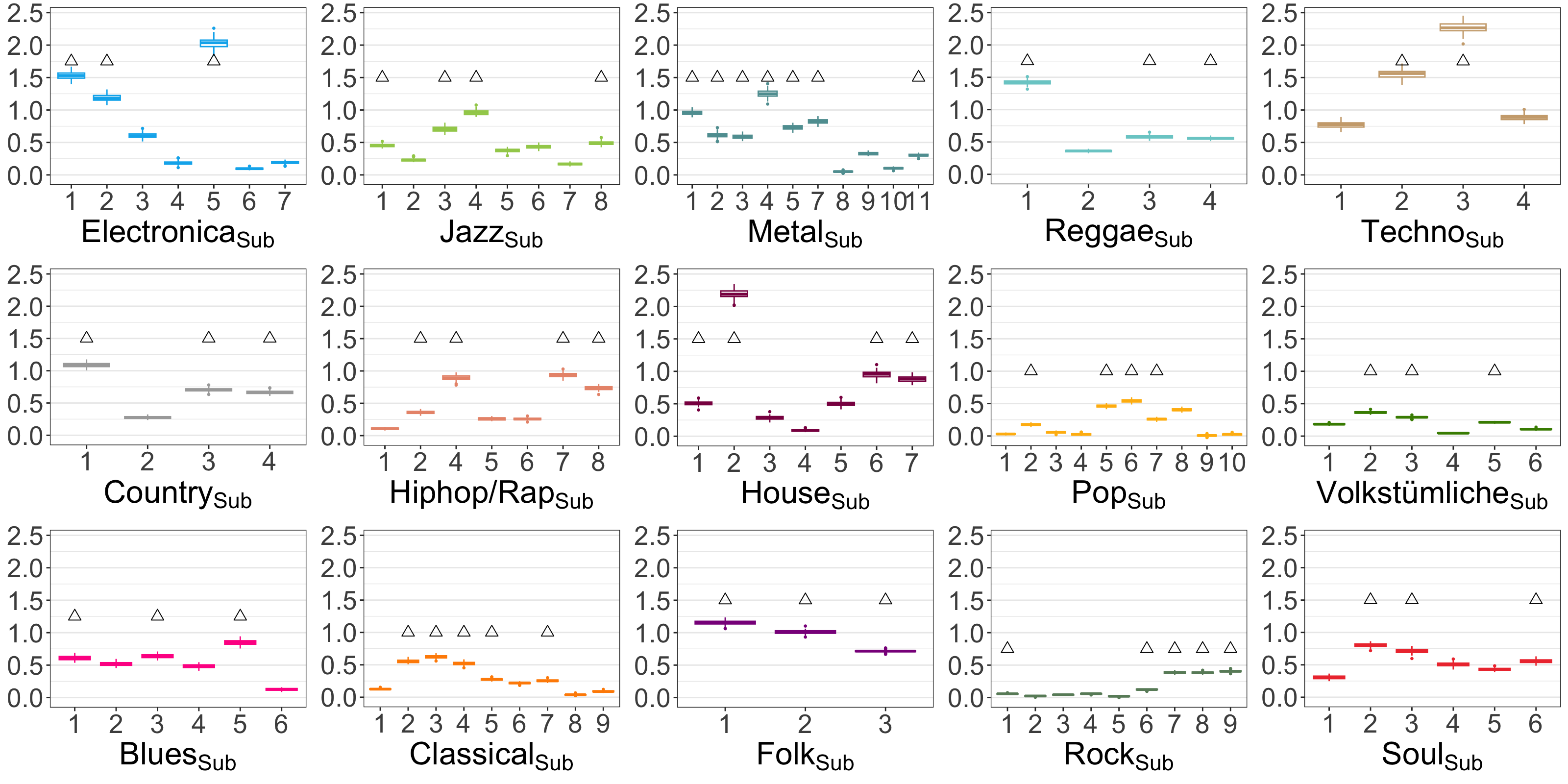

Supplement: Supplementary file 5 — Supporting Information. [file NYAS-1550-227-s001.png]
