# Supplementary material for: Categorizing music by genres
Source: Ann N Y Acad Sci. 2025 Jul 9;1550(1):227–38. doi: 10.1111/nyas.15404 (PMC12412717; doi:10.1111/nyas.15404)
Supplement: Supplementary file 6 — Supporting Information. [file NYAS-1550-227-s004.docx]

Supporting Materials for the manuscript

**Categorizing music by genre**

by

Elke B. Lange, Emily Gernandt, & Julia Merrill

accepted in

*Annals of the New York Academy of Sciences*

June 2025

We include here more detailed information and additional analyses for the interested reader. The serial order of this material is based on the main text:

Method: Materials

[**Table S1**](#TableS1)

Complete list of genre and subgenre categories, and artists listed as relevant for the subcategories.

Result section: Evaluation heuristics

[**Figure S1**](#FigureS1)

Percentage of “don’t know” responses of the subgenres embedded in the 15 genres.

[**Figure S2**](#FigureS2)

Percentage “don’t know” responses of the subgenres for two groups of participants.

Result section: Consistency of responses between genre and subgenres

[**Figure S3**](#FigureS3)

Visualization of distributions of liking ratings based on the genre-categories and M(sub) for all participants (row one and two) or participants grouped by their genre-ratings.

[**Table S2**](#TableS2)

Comparison on liking evaluations between category levels (based on subjects as cases).

[**Figure S4**](#FigureS4)

Ranking of genres by liking or percentage of unfamiliarity (% missing liking evaluation, “don’t know” responses) of genre-categories or mean across subgenre categories, M(sub), based on genres as cases (*N* = 15).

Result section: Homogeneity of liking subgenres from genres

[**Tables S3-5**](#TableS3)

Results of the 15 factor analyses, including the nested subgenres for each genre.

Result section: Typicality of exemplars

[**Table S6**](#TableS6)

Control measures for Figure 4, main text.

Result section: Representative exemplars for liking of genre

[**Table S7**](#TableS7)

Subgenre selection by random forest modeling.

[Added section: **Distinctiveness of taste dimensions: clarity of genre boundaries**](#DistinctivenessSection)

[**Figure S5.**](#FigureS5) Correlational structure of liking genre-categories (top row) or the average of subgenres, M(sub) (bottom row).

**Table S1.** Complete list of genre and subgenre categories, and artists listed as relevant for the subcategories.


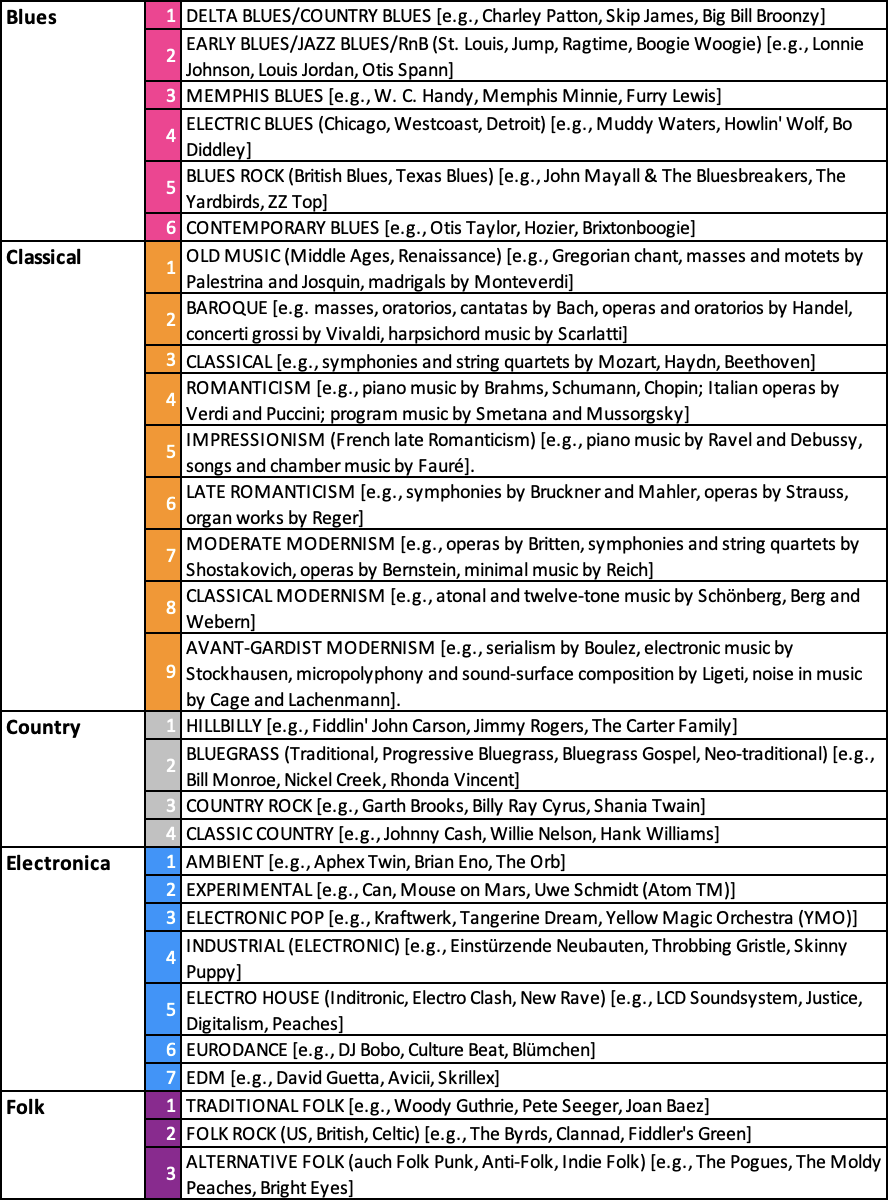


**Table S1** (continued)


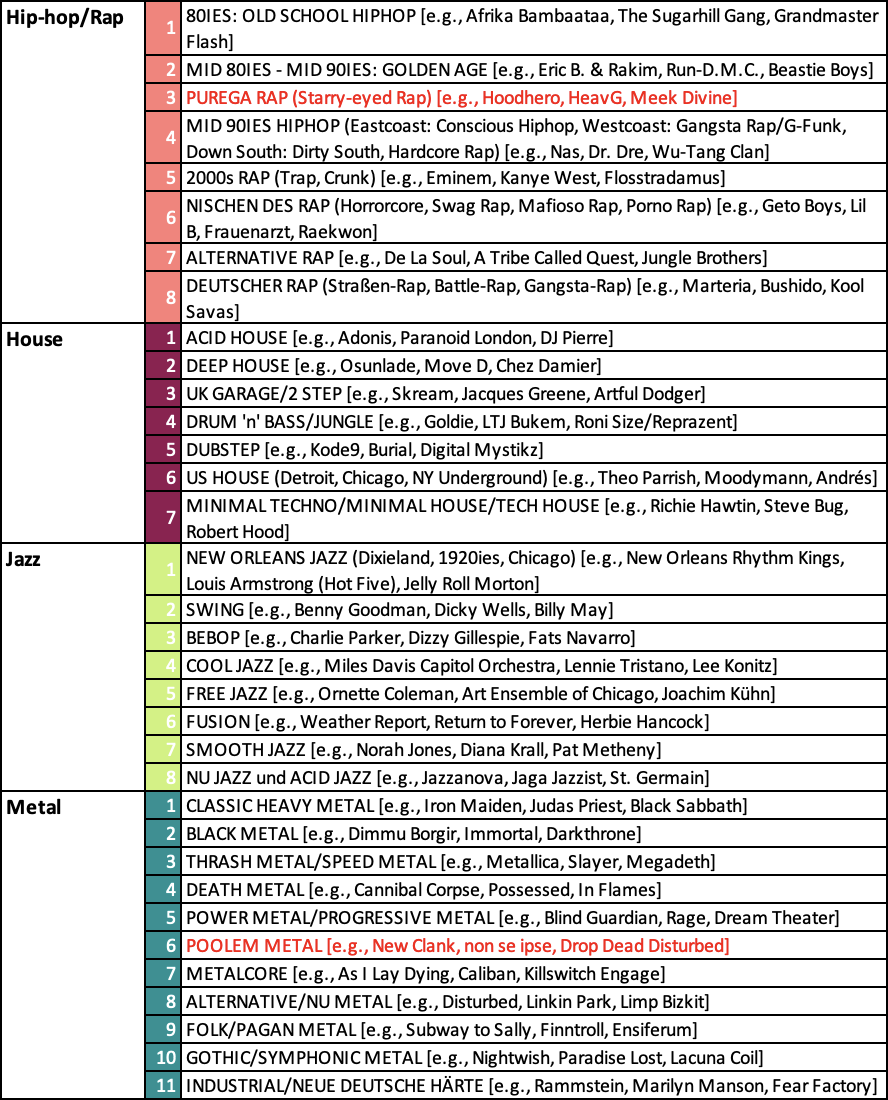


**Table S1** (continued)


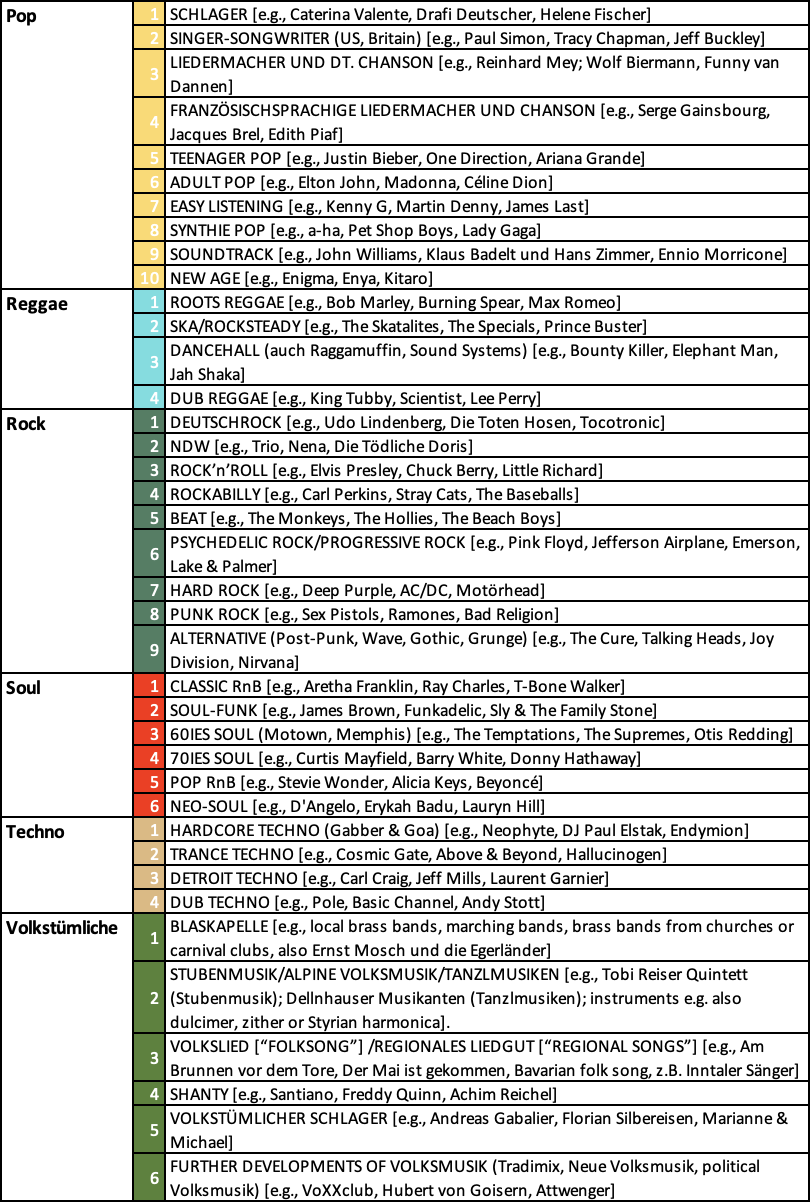


**Figure S1.** Percentage of “don’t know” responses of the subgenres embedded in the 15 genres.

**
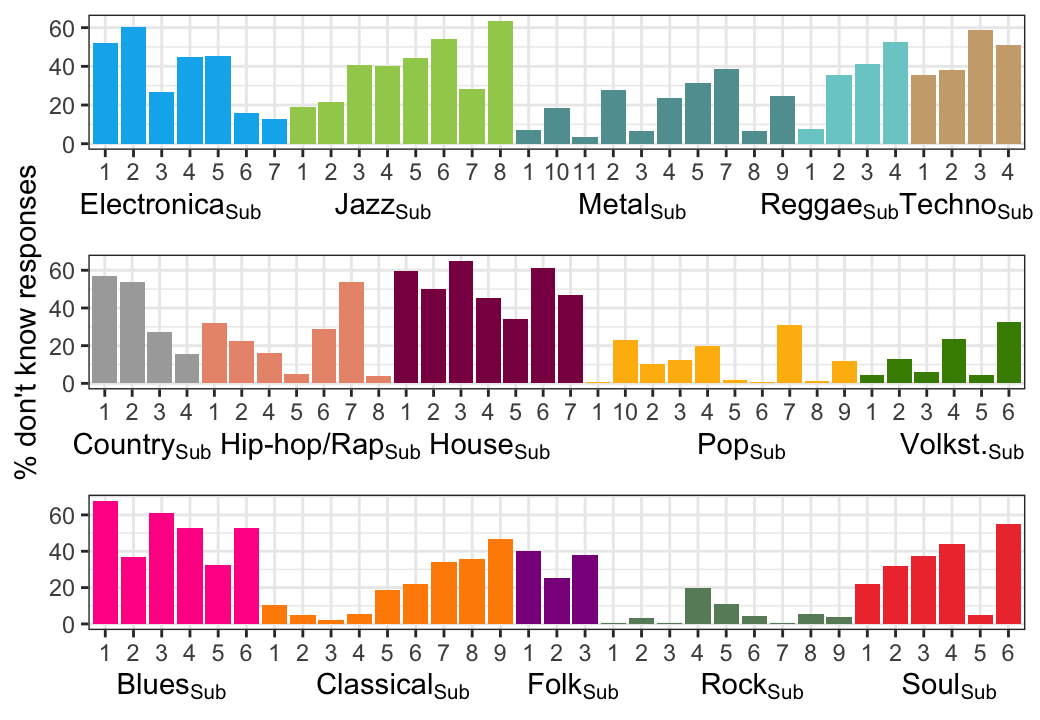
**

*Note*. The x-axis lists all subgenres, numbered from 1 to *n* for each genre, e.g., *electronica*_1_-*electronica*_7_ in the first row left-most, represent all seven subgenres for the genre *electronica*. The y-axis depicts the percentage of “don’t know this item” responses in the data set. Some genres’ styles were mostly unfamiliar (*house, blues*), others familiar (*pop, rock*), and the remaining showed a differentiated pattern across subgenres. For a complete list of subgenres and related musicians, see Table S1. Please compare this Figure S1 with the typicality of each subgenre for its genre (Figure 3, main text). Sometimes there is overlap (e.g., the most familiar *reggae* subgenre *reggae*_1_ more related to the genre *reggae* than the less familiar ones), sometimes not (the most familiar *blues* subgenres *blue*_6_ and *blue*_7_ have the lowest typicality for the genre *blues*).

**Figure S2.** Percentage “don’t know” responses of the subgenres for two groups of participants, plotted against each other and the diagonal added.

**
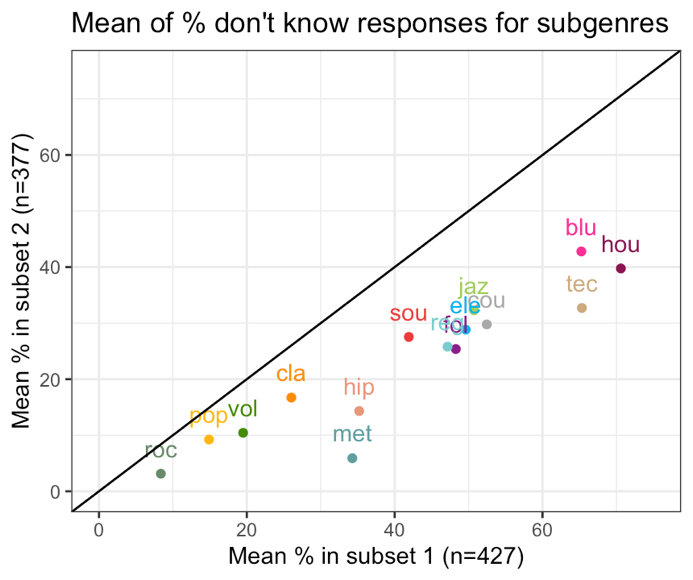
**

*Note.* From N=804 participants, only n=427 (subset_1) detected both foils (=gave the answer “don’t know this subgenre”). The remaining n=377 (subset_2) gave liking evaluations for at least one of the foils (i.e., the non-existing subgenres). One interpretation is that participants from subset_2 were less attentive than subset_1 when filling out the survey, and their responses might be less reliable. However, it is notable that the percentage of mean “don’t know” responses across subgenres is consistently higher for subset_1 than for subset_2 (the data points in Figure S2 are all below the diagonal with an intercept of zero and a slope of 1). This indicates that the subsets differed by their decision criteria to decide for “don’t know” responses. Participants from subset_1 responded more likely “don’t know”, when a subgenre was more unfamiliar to them, whereas participants from subset_2 responded more likely by an evaluation response following heuristics or based on associations with the label. As a result, chances are higher for subset_1 to respond “don’t know” than for subset_2 – also in the two cases of the foils (which were excluded in this Figure S2). Responses on foils, then, do not necessarily say much about the reliability of the participants.

**Figure S3.** Visualization of distributions of liking ratings based on the genre-categories and M(sub) for all participants (row one and two) or grouped by genre-ratings.


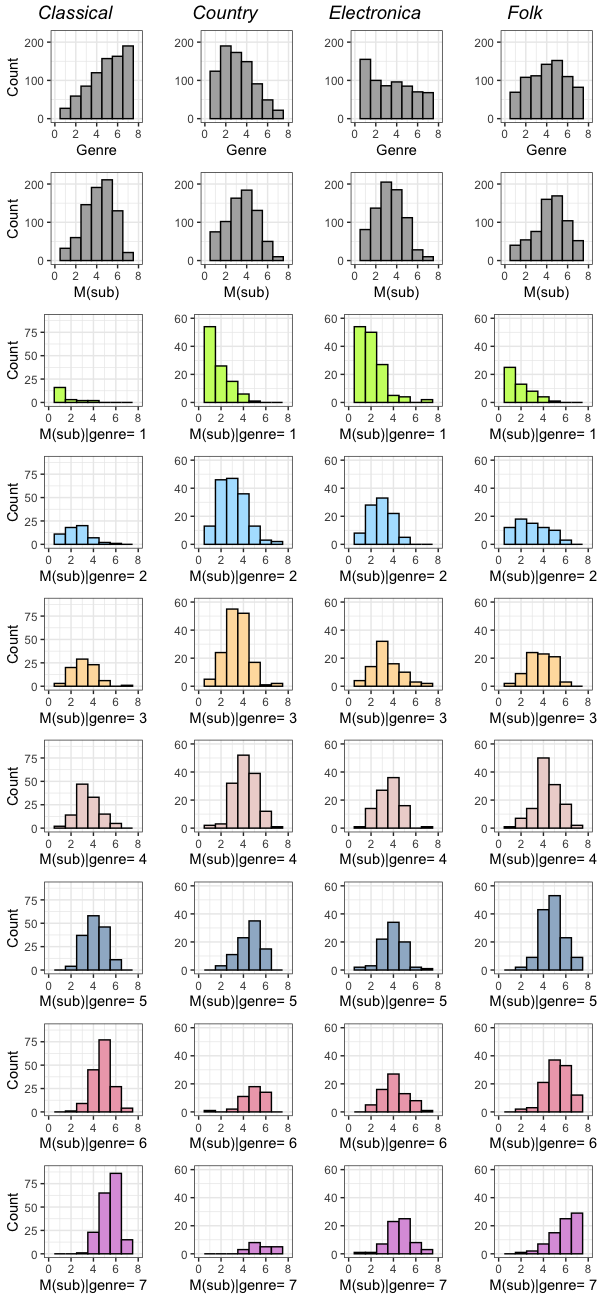


*Note.* The first two rows depict what has been shown in Figure 2 (main text) row one and three: distributions of ratings of genre-categories or mean across subgenres, M(sub). The subplots below depict specific groups of participants, based on their genre rating. For example, we selected participants included in the first column in row one (liking=1 for a specific genre like Classical, Country, Electronica, Folk) and show in row three their distribution of M(sub). In the next row, the distribution of M(sub) for participants from the second column in row one (liking=2) are shown and so on. That is, row three to last depict the subgenres means, grouped for participants differing in their genre rating from one to seven. All subplots show unimodal distributions. The means of those subplots move on the y-axis from lower to higher corresponding to the genre rating one to seven. For example, participants who selected “one” for the genre *Country*, show variability in their subgenres means (mostly ranging from one to four), but the distribution is unimodal with the peak at one. Similarly, participants who selected “two” for the genre *Country*, show a unimodal distribution with a low mean. The higher the selected rating for the genre (three to seven), the higher is the peak of the unimodal distribution. Further, the distribution of the genres is mirrored by the level of subplot distributions. For example, the distribution for the genre *Country* is right skewed, which corresponds to the observation that the peaks of the subplot distributions decrease from left to right. As another example, *Classical* has a left-skewed distribution for the genre ratings and the peaks across the subplots increase. That is, liking evaluations for a genre-label and related subgenres show variance but are systematically related.

**Table S2.** Comparison on liking evaluations between category levels (based on subjects as cases).

| Genre/  subgenres | *n* | *r* | *M_genre_* | *M_Msub_* | *t* | *p* | *d* |
| --- | --- | --- | --- | --- | --- | --- | --- |
| *Blues* | 660 | .68 | 4.386 | 4.364 | 0.473 | .636 | 0.02 |
| *Classical* | 789 | .62 | 4.992 | 4.244 | 18.396 | .000 | 0.65 |
| *Country* | 713 | .66 | 3.243 | 3.666 | -9.075 | .000 | -0.34 |
| *Electronica* | 636 | .61 | 3.574 | 3.353 | 3.377 | .001 | 0.13 |
| *Folk* | 651 | .64 | 4.333 | 4.407 | -1.398 | .163 | -0.05 |
| *Hip-hop/Rap* | 791 | .80 | 3.779 | 3.447 | 8.444 | .000 | 0.30 |
| *House* | 577 | .71 | 3.442 | 3.225 | 3.517 | .000 | 0.15 |
| *Jazz* | 739 | .69 | 4.39 | 4.078 | 6.543 | .000 | 0.24 |
| *Metal* | 789 | .82 | 4.025 | 3.558 | 9.572 | .000 | 0.34 |
| *Pop* | 802 | .59 | 4.539 | 3.485 | 21.662 | .000 | 0.76 |
| *Reggae* | 757 | .75 | 4 | 4.133 | -3.333 | .001 | -0.12 |
| *Rock* | 800 | .49 | 5.685 | 4.404 | 29.385 | .000 | 1.04 |
| *Soul* | 763 | .67 | 3.942 | 4.216 | -5.738 | .000 | -0.21 |
| *Techno* | 571 | .81 | 3.088 | 2.53 | 10.369 | .000 | 0.43 |
| *Volkst.* | 787 | .73 | 1.925 | 2.161 | -6.441 | .000 | -0.23 |

*Note:* Spearman correlation coefficients (*r*) between liking of genre-categories and mean across subgenres, M(sub), the subject-based mean liking based on genre ratings (M_genre_) or subgenres (M_sub_), and results of pairwise *t*-tests (with *df* = n-1) with uncorrected *p*-values (uncorrected *p* < 0.015 is equivalent to *p* < .05 before Bonferroni correction), and Cohen’s *d* for the paired t-test. Note that M_genre_ differs slightly from the means reported in Table 1 (main text), based on different numbers of cases.

**Figure S4.** Ranking of genres by liking or percentage of unfamiliarity (% “don’t know” responses) of genre-categories or mean across subgenre categories, M(sub), based on genres as cases (*N* = 15).

**Table S3.** Results of the factor analyses for nine genres for which the evaluations of related subgenres loaded on a single factor, indicating coherence between the evaluations of the subgenres.

|  | ***Blues*** | ***Country*** | ***Folk*** | ***House*** | ***Jazz*** | ***Reggae*** | ***Soul*** | ***Techno*** | ***Volkst.*** |
| --- | --- | --- | --- | --- | --- | --- | --- | --- | --- |
| 1 | 0.82 | 0.87 | 0.78 | 0.83 | 0.79 | 0.75 | 0.88 | 0.74 | 0.75 |
| 2 | 0.82 | 0.82 | 0.85 | 0.87 | 0.75 | 0.66 | 0.84 | 0.86 | 0.81 |
| 3 | 0.87 | 0.75 | 0.78 | 0.9 | 0.89 | 0.76 | 0.87 | 0.89 | 0.77 |
| 4 | 0.85 | 0.74 |  | 0.76 | 0.89 | 0.73 | 0.89 | 0.85 | 0.54 |
| 5 | 0.78 |  |  | 0.74 | 0.71 |  | 0.63 |  | 0.43 |
| 6 | 0.74 |  |  | 0.93 | 0.85 |  | 0.68 |  | 0.71 |
| 7 |  |  |  | 0.86 | 0.78 |  |  |  |  |
| 8 |  |  |  |  | 0.78 |  |  |  |  |
| observ. | 160 | 271 | 381 | 207 | 226 | 316 | 278 | 289 | 455 |
| %Var | 67 | 64 | 64 | 71 | 65 | 53 | 65 | 70 | 47 |
| RMSR | 0.05 | 0.02 | 0 | 0.07 | 0.08 | 0.05 | 0.08 | 0.06 | 0.07 |
| RMSEA | 0.16 | 0.046 |  | 0.209 | 0.232 | 0.171 | 0.210 | 0.322 | 0.149 |
| TLI | 0.904 | 0.994 | inf | 0.862 | 0.778 | 0.869 | 0.852 | 0.771 | 0.853 |
| CFI | 0.942 | 0.998 | 0.999 | 0.908 | 0.842 | 0.957 | 0.911 | 0.924 | 0.912 |

*Note*. The number of genre-related subgenres differed from three (*folk*) up to eight (*jazz*) in these examples. Observ.: Number of observations, % Var: Percentage of explained variance, RMSR: Square root of the mean of the squared residuals as an index of the overall badness-of-fit, RMSEA: Root mean square error of approximation as an absolute fit index [with values of 0.01 excellent and 0.08 mediocre fits], TLI: Tucker-Lewis index [>.90 acceptable], CFI: comparative fit index [> .95 good]. *Volkst*.: “*Volkstümlich*”[dt.].

**Table S4.** Results of the factor analyses for *classical, electronica*, and *hip-hop/rap* for which the evaluations of related subgenres loaded on more than a single factor.

|  | ***Classical*** | | ***Electronica*** | | ***Hip-hop/Rap*** | |
| --- | --- | --- | --- | --- | --- | --- |
|  | Factor A | Factor B | Factor A | Factor B | Factor A | Factor B |
| 1 | **0.49** | 0.09 | **0.90** | -0.05 | **0.93** | -0.07 |
| 2 | **0.78** | -0.03 | **0.96** | -0.15 | **0.88** | -0.02 |
| 3 | **0.90** | -0.16 | **0.78** | 0.13 | foil |  |
| 4 | **0.88** | -0.005 | **0.75** | 0.08 | **0.60** | 0.4 |
| 5 | **0.71** | 0.21 | **0.73** | 0.22 | 0.28 | **0.62** |
| 6 | **0.72** | 0.15 | 0.06 | **0.68** | 0.02 | **0.7** |
| 7 | **0.55** | 0.4 | -0.02 | **0.71** | **0.47** | 0.39 |
| 8 | 0.07 | **0.85** |  |  | -0.09 | **0.92** |
| 9 | -0.05 | **0.92** |  |  |  |  |
| observ. | 337 | | 238 | | 278 | |
| %Var | 44 | 22 | 50 | 16 | 36 | 32 |
| RMSR | 0.05 | | 0.02 | | 0.03 | |
| RMSEA | 0.147 | | 0.105 | | 0.148 | |
| TLI | 0.868 | | 0.944 | | 0.895 | |
| CFI | 0.931 | | 0.979 | | 0.960 | |

*Note*. The number of genre-related subgenres differed with up to nine for Classical in these examples. Observ.: Number of observations, % Var: Percentage of explained variance, RMSR: Square root of the mean of the squared residuals as an index of the overall badness-of-fit, RMSEA: Root mean square error of approximation as an absolute fit index [with values of 0.01 excellent and 0.08 mediocre fits], TLI: Tucker-Lewis index [>.90 acceptable], CFI: comparative fit index [> .95 good].

**Table S5.** Results of the factor analyses for *metal, pop*, and *rock* for which the evaluations of related subgenres loaded on more than a single factor.

|  | ***Metal*** | | ***Pop*** | | ***Rock*** | | |
| --- | --- | --- | --- | --- | --- | --- | --- |
|  | Factor A | Factor B | Factor A | Factor B | Factor A | Factor B | Factor C |
| 1 | **0.74** | 0.04 | **0.40** | 0.11 | 0.18 | 0.09 | **0.56** |
| 2 | **0.91** | -0.15 | 0.16 | **0.55** | -0.04 | -0.01 | **0.89** |
| 3 | **0.84** | -0.02 | 0 | **0.77** | 0.01 | **0.88** | -0.03 |
| 4 | **0.86** | 0 | -0.01 | **0.67** | -0.04 | **0.84** | 0.01 |
| 5 | **0.77** | 0.13 | **0.72** | -0.22 | 0.1 | **0.55** | 0.13 |
| 6 | foil |  | **0.8** | 0.06 | **0.58** | 0.14 | 0 |
| 7 | **0.4** | 0.38 | **0.67** | 0.05 | **0.7** | 0.04 | -0.06 |
| 8 | -0.07 | **0.84** | **0.7** | 0.03 | **0.81** | -0.01 | 0.04 |
| 9 | **0.56** | 0.32 | **0.42** | 0.21 | **0.74** | -0.07 | 0 |
| 10 | **0.46** | 0.44 | **0.62** | 0.06 |  |  |  |
| 11 | 0.29 | **0.58** |  |  |  |  |  |
| observ. | 401 | | 390 | | 573 | | |
| %Var | 45 | 19 | 29 | 15 | 24 | 21 | 13 |
| RMSR | 0.05 | | 0.05 | | 0.04 | | |
| RMSEA | 0.165 | | 0.103 | | 0.154 | | |
| TLI | 0.825 | | 0.850 | | 0.747 | | |
| CFI | 0.899 | | 0.914 | | 0.916 | | |

*Note.* The number of genre-related subgenres differed with up to eleven for Metal in these examples. Observ.: Number of observations, % Var: Percentage of explained variance, RMSR: Square root of the mean of the squared residuals as an index of the overall badness-of-fit, RMSEA: Root mean square error of approximation as an absolute fit index [with values of 0.01 excellent and 0.08 mediocre fits], TLI: Tucker-Lewis index [>.90 acceptable], CFI: comparative fit index [> .95 good].

**Table S6.** Control measures for Figure 4, main text.

| Subgenres from | *M* | *SD* | Median | *M* + 1*SD* |
| --- | --- | --- | --- | --- |
| Blues | 0.06 | 0.07 | 0.04 | 0.13 |
| Classical | 0.02 | 0.03 | 0.01 | 0.05 |
| Country | 0.04 | 0.05 | 0.01 | 0.08 |
| Electronica | 0.04 | 0.07 | 0.01 | 0.11 |
| Folk | 0.03 | 0.04 | 0.02 | 0.07 |
| Hip-hop/Rap | 0.03 | 0.04 | 0.01 | 0.07 |
| House | 0.07 | 0.14 | 0.01 | 0.21 |
| Jazz | 0.06 | 0.07 | 0.03 | 0.13 |
| Metal | 0.02 | 0.04 | 0.00 | 0.06 |
| Pop | 0.03 | 0.04 | 0.01 | 0.07 |
| Reggae | 0.03 | 0.04 | 0.02 | 0.07 |
| Rock | 0.04 | 0.06 | 0.01 | 0.10 |
| Soul | 0.05 | 0.07 | 0.02 | 0.12 |
| Techno | 0.06 | 0.13 | 0.01 | 0.19 |
| Volkstueml. | 0.02 | 0.02 | 0.01 | 0.04 |

*Note.* For each subset of nested subgenre, we report the mean *r*^2^, its SD and the median *r*^2^. The line in Figure 4 depicts the *M* + 1 *SD*.

**Table S7.** Subgenre selection by random forest modeling

| Genre/  subgenres | *n* | *threshold* | *interpretation* | *prediction* |
| --- | --- | --- | --- | --- |
| Blues | 159 | 5 3 1 2 4 6 (all) | 5 3 1 2 4 | 5 3 1* |
| Classical | 337 | 3 2 4 5 7 6 1 9 8 (all) | 3 2 4 5 7* | 3 2 4 5 7 |
| Country | 271 | 1 3 4 2 (all) | 1 3 4* | 1 3 4 |
| Electronica | 235 | 5 1 2 3 7 4 6 (all) | 5 1 2 3 7 | 5 1 2* |
| Folk | 381 | 1 2 3* (all) | 1 2 3 | NULL |
| Hip-hop/Rap | 278 | 7 4 8 2 5 6 1 (all) | 7 4 8 2* | 7 4 8 2 |
| House | 207 | 2 6 7 1 5 3 4 (all) | 2 6 7 1 5 | 2 6 7 1* |
| Jazz | 226 | 4 3 8 1 6 5 2 7 (all) | 4 3 8 1* | 4 3 8 1 |
| Metal | 399 | 4 1 7 5 2 3 9 11 10 8 (all) | 4 1 7 5 2 3 9 11 | 4 1 7 5 2 3 11* |
| Pop | 390 | 6 5 8 7 2 3 1 4 10 (all but 9) | 6 5 8 7 2 | 6 5 7 2* |
| Reggae | 316 | 1 3 4 2 (all) | 1 3 4* | 1 3 4 |
| Rock | 569 | 9 8 7 6 1 4 3 2 5 (all) | 9 8 7 6 1 4 | 9 8 7 6 1* |
| Soul | 276 | 2 3 6 4 5 1 (all) | 2 3 6 | 2 3 6* |
| Techno | 288 | 3 2 4 1 (all) | 3 2* | 3 2 |
| Volkstueml. | 455 | 2 3 5 1 6 4 (all) | 2 3 5* | 2 3 5 |

*Note.* Results of the subgenre selection by VSURF (Genuer, 2015) based on three selection criteria, taking into account (a) mean relative importance and its standard deviation (threshold), (b) as well as out-of-bag (OOB) error, and (c) based on a and b being more conservative. Serial orders of subgenre indices are based on their relative importance within the model. *denotes the final selection, which was “interpretation”, if prediction did not further reduce the selection.

**Added section:**

**Distinctiveness of taste dimensions: clarity of genre boundaries**

Finally, we report here a change of perspective and explored latent factors of taste dimensions, that is, we nested the genres in even broader categories. We performed two factor analyses to uncover underlying dimensions of musical taste: one factor analysis with the genre ratings as items, and the other with the M(sub) as items.^[[1]](#footnote-1)^ The solutions were interpreted based on model fit, number and plausibility of factors. We deleted participants with missing liking ratings on the genre level, resulting in n = 617 and n = 358 cases.^[[2]](#footnote-2)^ We calculated the Kaiser-Meyer-Olkin test, showing that the set with genre categories was less correlated (measure of sampling adequacy, MSA = .71) than the M(sub) set (MSA = .83). However, for the genre set, *Pop* MSA < .50, indicating that the item *Pop* was better excluded from the factor analysis, which we did for both sets accordingly. Figures S5 A and D compare the pairwise Spearman rank correlation coefficients for the genre set (upper panel) and the M(sub) set (lower panel; *Pop* included). The subplots visualize hierarchical clustering (*corrplot* in R, Wei & Simko, 2021), showing distinct clusters in the genre set in comparison to the M(sub) set. The Scree-plot for both sets (*Pop* excluded) indicated a more differentiated structure of five factors for the genre set (Figure S5 B), than the three factors for the M(sub) set (Figure S5 E). However, when we fitted the factor analyses, for genre, the five factors resulted in a sufficient solution (empirical Χ^2^ = 31.8 , *p* < .46, TLI = .956, RMSEA = .046), whereas for the M(sub) set the three factors did not result as well as the four factors (*p* < .0001). Again, the five factors solution was sufficient in the M(sub) set (empirical Χ^2^ = 34.6 , *p* < .30, TLI = .931, RMSEA = .065). Both solutions are statistically sound, factor loadings are higher for the M(sub) solution. The categories within the genre-set (Figure S5 C) are clustered in a reasonable way and dimensions converge with what is known by the literature, whereas this is less the case for the factor analysis on M(sub) (Figure S5 F).

The conclusion here is that M(sub) has mathematical properties that might forestall a clear differentiation into upper-level taste dimensions. Averaging across subgenre-ratings makes the distributions of M(sub) more similar to each other (unimodal or normal), and therefore, splitting the data into higher-level dimensions is more difficult. In addition, it is likely that evaluations on the genre level include not only evaluations on the singular items but also relations of liking between the genres, whereas evaluations on the subgenre level might be comparative between the nested subgenres but not between subgenres from different genres. Then, taste dimensions might be better evaluated on a limited set of genre-categories than on a multitude of subgenres. Note, that the high percentage of “don’t know” responses prevented a factor analysis taking all subgenres into account at the same time. Only 27 participants gave liking ratings for all 100 subgenres. Taking M(sub) as items was a solution to consider evaluations on subgenre levels nevertheless. However, as we have argued, this solution is not ideal and introduces other problems.


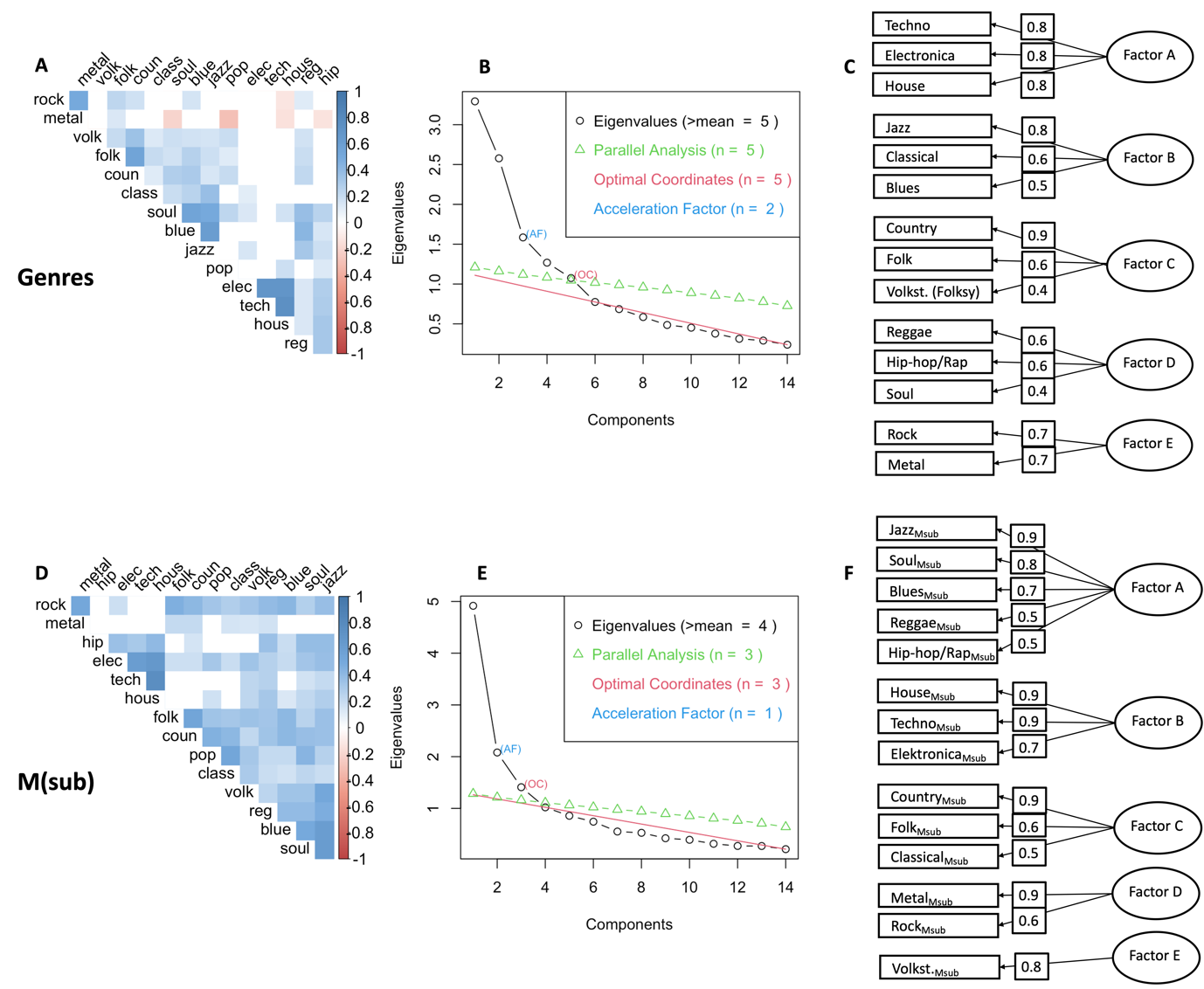


**Figure S5.** Correlational structure of liking genre-categories (top row) or the average of subgenres, M(sub) (bottom row). A, D: Visualization of pairwise correlations, the genre labels are abbreviated by the first 3 or 4 letters (applying the function *corrplot* in R, Wei & Simko, 2021). B, E: Screeplots of eigenvalues (Raiche et al., 2013). C, F: Five factor solutions of the factor analyses.

**References**

Bernaards, C.A., & Jennrich, R.I. (2005). Gradient projection algorithms and software for arbitrary rotation criteria in factor analysis. Educational and Psychological Measurement, 65, 676–696. https://doi.org/10.1177/0013164404272507

Raiche, G., Walls, T. A., Magis, D., Riopel, M. and Blais, J.-G. (2013). Non-graphical solutions for Cattell's scree test. *Methodology, 9*(1), 23-29. https://doi.org/10.1027/1614-2241/a000051

Revelle, W. (2024). psych: Procedures for Psychological, Psychometric, and Personality Research. Northwestern University, Evanston, Illinois. R package version 2.4.6, https://CRAN.R-project.org/package=psych

Wei, T., & Simko, V. (2021). *R package 'corrplot': Visualization of a Correlation Matrix*. (Version 0.92), https://github.com/taiyun/corrplot

1. For genres: Extraction method was a principal axis factor because of non-normally distributed data, and oblique rotation, as factors were correlated. For M(sub): We calculated maximum likelihood because of normally distributed data, and again oblique rotation. Both analyses were run by the function *fa*() in R from the *psych* library (Revelle, 2024), rotation = promax by the *GPArotation* package in *R* (Bernaards & Jennrich, 2005). [↑](#footnote-ref-1)
2. The M(sub) ignores don’t know responses (missing cases) on the subgenre level. We calculated an unweighted average across the remaining liking responses. [↑](#footnote-ref-2)
